# Soft Nanoonions: A Dynamic Overview onto Catanionic Vesicles Temperature-Driven Transition

**DOI:** 10.3390/ijms21186804

**Published:** 2020-09-16

**Authors:** Gesmi Milcovich, Filipe E. Antunes, Mario Grassi, Fioretta Asaro

**Affiliations:** 1 Department of Chemical and Pharmaceutical Sciences, University of Trieste, via L. Giorgieri 1, 34127 Trieste, Italy; gesmi.milcovich@gmail.com (G.M.); fasaro@units.it (F.A.); 2 School of Chemical Sciences, Dublin City University (DCU), Dublin 9, Ireland; 3 Coimbra Chemistry Centre, Department of Chemistry, University of Coimbra, 3004-535 Coimbra, Portugal; filipe.antunes@ci.uc.pt; 4 Department of Engineering and Architecture, University of Trieste, via A. Valerio 6/A, 34127 Trieste, Italy

**Keywords:** catanionic vesicles, diffusion, NMR, nanoreservoirs, microscopy

## Abstract

Catanionic vesicles are emerging interesting structures for bioapplications. They self-generate by a pairing of oppositely charged ionic surfactants that assemble into hollow structures. Specifically, the anionic-cationic surfactant pair assumes a double-tailed zwitterionic behavior. In this work, the multilamellar-to-unilamellar thermal transition of several mixed aqueous systems, with a slight excess of the anionic one, were investigated. Interestingly, it was found that the anionic counterion underwent a dissociation as a consequence of a temperature increase, leading to the mentioned thermal transition. The present work proposed the spectroscopic techniques, specifically multinuclear NMR and PGSTE (pulsed gradient stimulated echo), as a key tool to study such systems, with high accuracy and effectiveness, while requiring a small amount of the sample. The results presented herein evidence encouraging perspectives, forecasting the application of the studied vesicular nanoreservoirs, for e.g., drug delivery.

## 1. Introduction

Catanionic vesicles can be easily developed, thanks to the self-assembly of oppositely charged ionic amphiphiles, leading to the formation of colloidal hollow structures. Indeed, a double-tailed zwitterionic behavior is responsible for the pairing of the anionic-cationic surfactants. Therefore, the above-mentioned zwitterionic double-chained structure self-generates by the association of oppositely charged single-tailed surfactants [1,2,3]. Nowadays, these vesicular systems are of increasing interest as they are widely employed in the pharmaceutical/biotechnological field (e.g., targeted gene therapy, medicated syrups, eye drop products, etc.) [4,5,6,7]. They mimic biological membranes and their related compartmentalization properties, while noteworthily, their preparation is quite cheap and easy [1,8]. Catanionic mixed systems tend to spontaneously aggregate into multi-walled vesicular structures [8], specifically in the presence of a slight excess of the anionic counterpart [9]. Those mixtures can undergo a multi-to-unilamellar transition due to different parameters’ changes, e.g., temperature, salt/co-solutes addition, and surfactant chain length [10,11]. Thus, the mentioned transition can be triggered by a temperature increase, which enhances the anionic counterion dissociation.

In this work, spectroscopic techniques (NMR, UV-Vis), fluorescence microscopy, and polarized light microscopy were used to further understand the dynamics of the temperature effects. Particularly, a powerful NMR experimental setup was applied, namely PGSTE (pulsed gradient stimulated echo), which is able to discriminate signals for multicomponent mixtures spectra, taking advantage of a different diffusion attitude of the molecular species [12]. In addition, another useful NMR tool for biological/chemical investigation used therein was the transverse relaxation rate R_2_ [13]. The sodium transverse relaxation rates (^23^Na-R_2_) were studied, as Na^+^ corresponds to the counterion of the anionic component in excess. The ^23^Na isotope was characterized by a high NMR sensitivity, a spin of I = 3/2, and a natural abundance of 100%. Therefore, the quadrupolar mechanism was mainly responsible for the rule out of its relaxation, and the self-assembly properties of catanionic systems could be easily studied by the NMR dynamic parameters [14,15]. In terms of approach, the following comparison sequence sodium dodecyl sulfate/cetyltrimethylammonium bromide (*SDS*/*CTAB*) > *SDS*/cetylpyridinium bromide (*CPB*) > sodium decyl sulfate (SdS)/*CPB* was used, bearing in mind their molecular solubility scales and their critical micellar concentration (See Table 1 below).

Therefore, these kinds of systems and their investigation through NMR techniques propose an elegant approach to encompass the use of a very small amount of the bioactive molecules to be included, both for basic research and future scale-up. The study and optimization of these nanoreservoirs are of increasing interest for the scientific community, thanks to several advantages they can provide for different applications, such as the drug delivery sector. The preparation of the cationic vesicles is quick and simple, the raw materials required are not expensive, and the nanoreservoirs can be used either on their multilamellar or their unilamellar form due to their irreversible thermal transition. Furthermore, their soft nature allows them to include both hydrophilic and hydrophobic bioactive molecules. To the best of our knowledge, this report represents the first strategic approach of multinuclear NMR applied as a powerful tool to optimize and study soft nanoshuttles for innovative use in the future use in the bioactive molecules delivery field.

## 2. Results and Discussion

### 2.1. SDS/CTAB System

**Figure 1:**
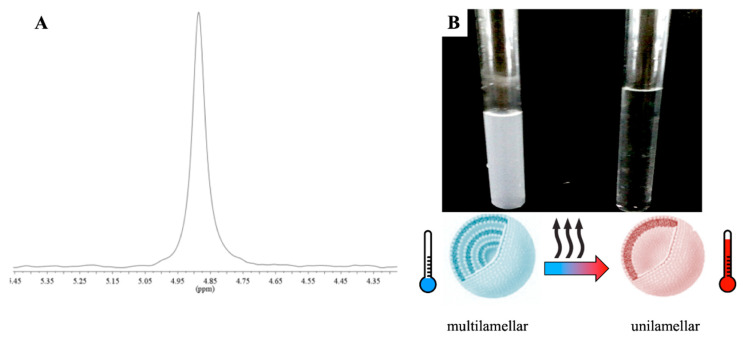
Multi-to-unilamellar vesicles thermal transition. (**A**) ^2^H-NMR spectrum of *SDS*/*CTAB* aqueous solution and (**B**) decrease of turbidity with heating.

A constant C_TOT_ = 6 mM was kept (i.e., about 0.2% wt) in aqueous solution, whereas the surfactant molar ratios (R) were changed (see Materials and Methods Section). According to the literature and to obtain a fluid-like state for the alkyl chains, a temperature slightly higher than 25 °C was set. To avoid multiple phases/micelles in vesicular samples, highly diluted solutions were used, as previously mentioned (see Materials and Methods, Section 3.1). With reference to Equation (1), catanionic vesicles effectively self-assembled at *R* ≠ 1 (negatively or positively). The focus of the present experimental work involved just the anionic side of the phase diagram, as both at *R* = 1 and in the cationic region of the ternary phase diagram led to precipitation (see Materials and Methods, Section 3.1). Moreover, vesicles solutions with *R* = 1 exhibit a Krafft point above 100 °C [19], which makes them not suitable for biological applications. Single-walled nanoreservoirs kept their unilamellar state for months, should a temperature close to 25.0 °C be provided. Otherwise, phase separation could occur due to the *CTAB* Krafft point, which lied in the 25–27 °C range, as mentioned in Materials and Methods, Section 3.1. Considering that a negative charge allows vesicles to be stable, an excess of sodium dodecyl sulfate was used, i.e., *R* > 1 (see Equation (1), Materials and Methods Section). Vesicular solutions appeared milky as soon as they self-stabilized due to the presence of multilamellar structures. On the other hand, straight after the thermal transition, their characteristic turbidity completely vanished (Figure 1), leading to their conversion into unilamellar aggregates, with a lower hydrodynamic radius [8]. The thermal transition was found to be irreversible within the 46–47 °C thermal range for catanionic vesicles characterized by an *R* = 1.7 and *R* = 1.85, respectively. Anisotropic lamellar phases occurrence had been ruled out, both prior and after the thermal transition, due to a lack of the water signal splitting in the ^2^H-NMR spectrum (see below Figure 1). The mentioned finding was also confirmed by the complete absence of birefringence in the polarized light microscopy observations.

**Figure 2:**
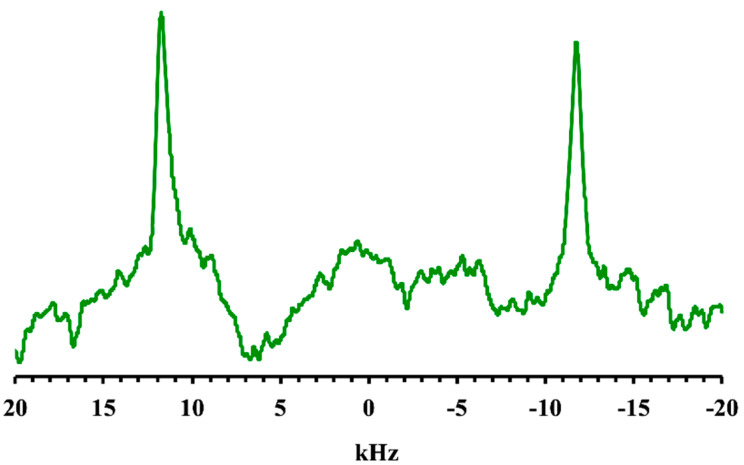
^14^N-NMR spectrum of *SDS*/*CTAB* aqueous solution (*R* = 1.5 sample).

The ^14^N NMR spectrum (^14^N nucleus has I = 1) of the tetraalkylammonium head group provided for consistent information into the bilayers ordering, as it refers to the molecular moiety with the lowest mobility (*CTAB*). The residual quadrupolar splitting (23.5 kHz) could be compared to the same splitting, which defines diluted lyotropic liquid crystals (Figure 2) [20]. The line width was rather small due to an orientational effect caused by the magnetic field, as already known for liposomes [21,22].

**Figure 3:**
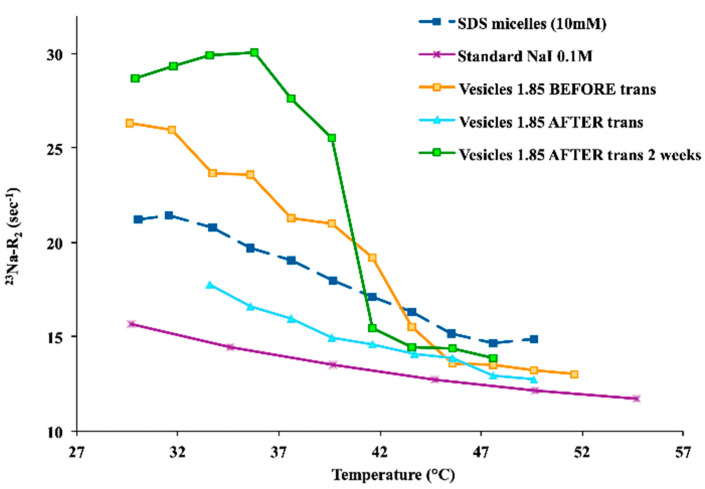
^23^Na-R_2_ values for 1.85 *SDS*/*CTAB* vesicular solutions (before and after thermal transition), compared with a standard reference solution of NaI (free Na^+^) and a micellar solution of *SDS*.

On the other hand, longitudinal relaxation rates, R_1_, were remarkably less sensitive to slow motions than ^23^Na transverse relaxation rates, R_2_. The echo decay complied with a single exponential both before and after the thermal transition. No significant deviations from the single exponential were present, according to the application of very diluted catanionic systems. Therefore, R_2_ values refer to averages related to central and satellite transitions [23]. By comparing the ^23^Na R_2_ trends, it was possible to observe a remarkable difference within the free Na^+^, with reference to the negatively charged vesicles. Indeed, the interaction of the counterion with negatively charged catanionic vesicles showed to be higher than for free Na^+^ (e.g., NaI 0.1 M standard solution). Conversely, the ^23^Na R_1_ trends did not demonstrate a significant difference among the catanionic aggregates and the free Na^+^. Moreover, higher molar ratios R resulted in larger ^23^Na R_2_ values. A ^23^Na R_2_ decrease was observed with increasing temperature, reaching the ^23^Na R_2_ range values for the standard free Na^+^, after the critical transition temperature (Figure 3). Therefore, the ^23^Na R_2_ values decreased when the temperature increase was consistent with the main dissociation of Na^+^ from the vesicular aggregates. Additionally, the mentioned phenomena affected the packing parameter, which was the driving force for the spontaneous interfacial curvature. This was due to a screening decrease of the repulsive interactions among head groups of the same charge, affecting the tail mobility, as well as enhancing the related volume.

**Figure 4:**
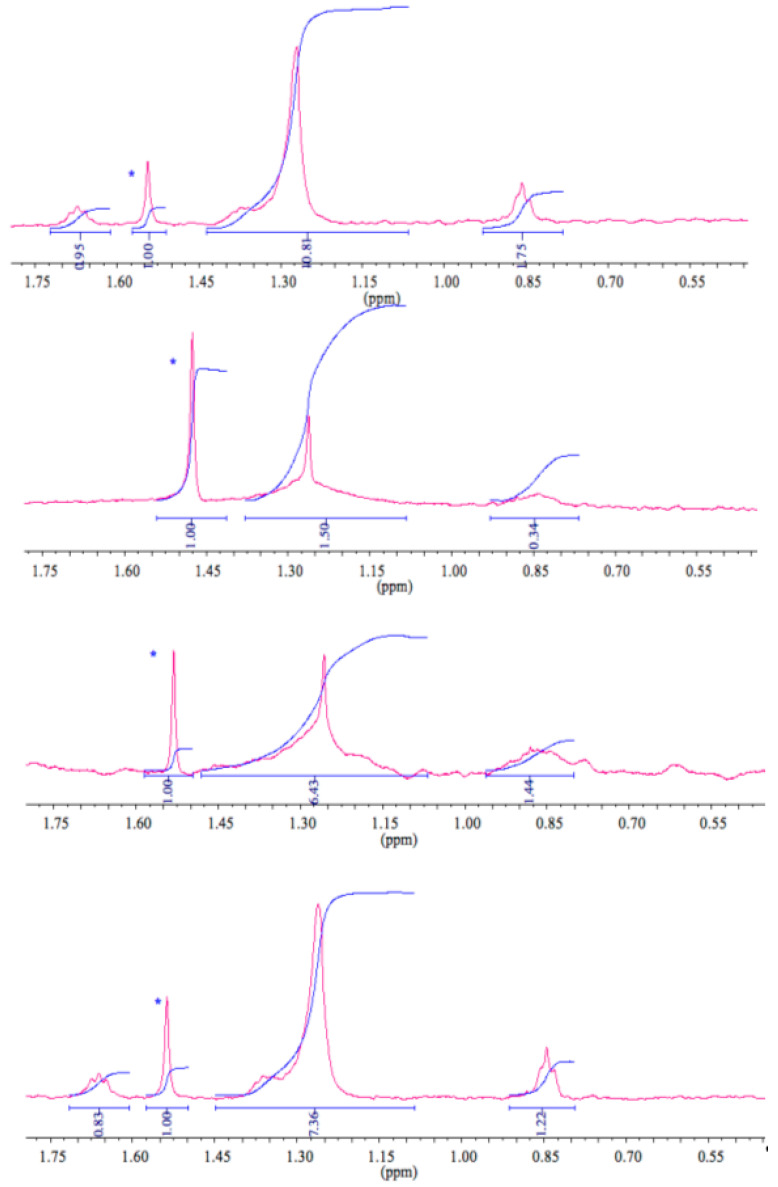
^1^H-NMR spectra of an *SDS*/*CTAB* aqueous solution with integration. From bottom to top: 30 °C, 50 °C, 30 °C immediately after transition, and 30 °C two weeks after the transition. Peaks of water included in CDCl_3_ are marked with *.

Interestingly, when the ^23^Na R_2_ measurements were acquired some days after samples were brought back to room temperature, higher ^23^Na R_2_ values were recorded (especially for the higher molar ratio R samples) due to changes in the correlation times of the motions modulating the quadrupolar interaction. The lower sodium dissociation led the process, confirming the rise in the dodecyl sulfate vesicles content after the transition, as endorsed by the ^1^H-NMR spectra (Figure 4). As expected, all detectable ^1^H-NMR signals corresponded to the monomeric anionic surfactant, showing no signal for the cationic counterpart. The faster dodecyl sulfate exchange among bulk and vesicles was responsible for the line width broadening for the ^1^H dodecyl sulfate resonances, together with the heating cycle. According to the ^23^Na R_2_ values, ^1^H-NMR integrals analyses indicated lower values at high temperatures too. These findings demonstrated the dynamic uptake of the anionic component into the vesicular aggregates, confirming a higher dodecyl sulfate amount in the composition of the vesicles.

**Figure 5:**
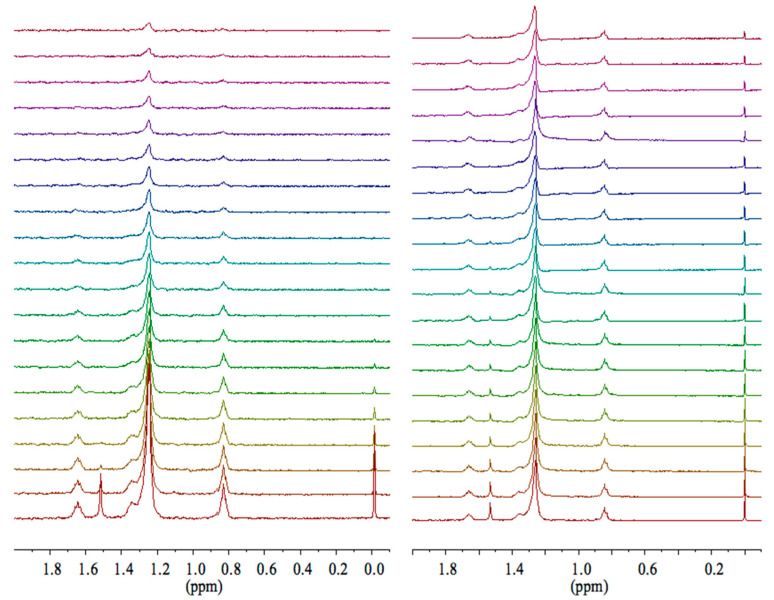
PGSTE-NMR spectra of 1.85 molar ratio *SDS*/*CTAB* solutions, collected at 30 °C, with Δ = 60 ms (**left**) and Δ = 10 ms (**right**). PGSTE, pulsed gradient stimulated echo.

**Figure 6:**
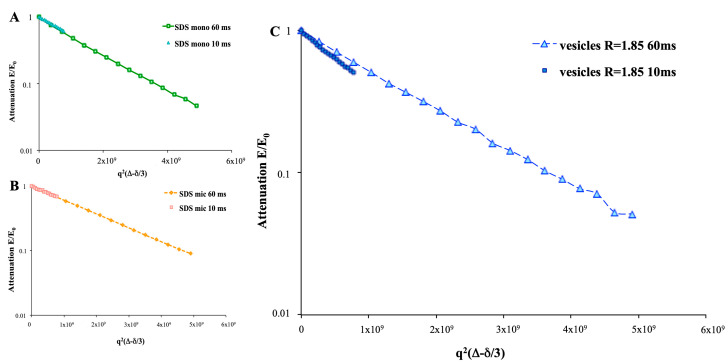
Plot of the echo decays at different diffusion intervals at 30 °C (Δ = 10 ms, Δ = 60 ms) for (**A**) an *SDS* monomeric solution, (**B**) *SDS* micellar solution (10 mM), and (**C**) vesicles at *R* = 1.85.

The isotropy of the solutions was endorsed by the application of PGSTE experiments at different diffusion intervals and related analysis for the trends of the echo intensity: no shift changes were observed at the strongest gradients (Figure 5). Moreover, by plotting PGSTE echo decays at different diffusion delays (Δ = 10 ms, Δ = 60 ms) for vesicular samples, an interesting slope difference was detected, whereas no slope deviations were present both in monomeric and micellar dodecyl sulfate samples. A slow exchange (~3 Hz) between the vesicular aggregates and the bulk was noticed for the dodecyl sulfate in vesicles. On the other hand, a fast exchange (10^4^ Hz) with the bulk in micellar samples was noticed for the dodecyl sulfate. According to Johnson [12], the PGSTE data, at different delta intervals (Δ), demonstrated a dependence of the echo decays on delta intervals (Δ in vesicular solutions). Viceversa, no relationship among echo decays and diffusion delays were detected for both micellar and monomeric dodecyl sulfate samples (Figure 6).

**Figure 7:**
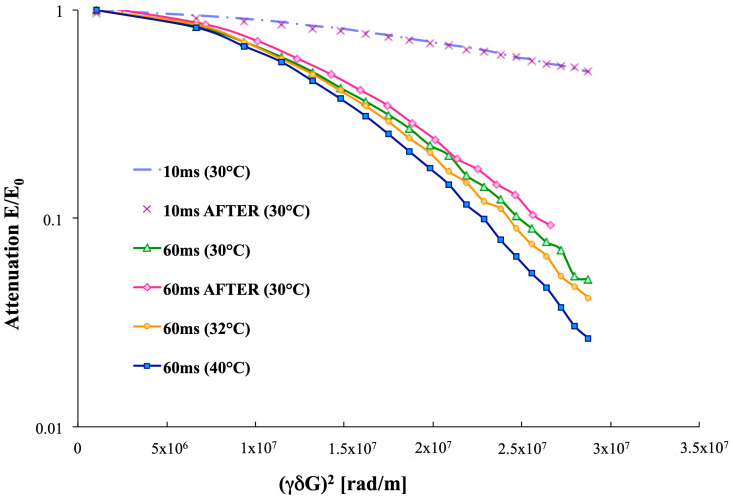
The plot of the echo decays at different diffusion intervals (Δ = 10 ms, Δ = 60 ms) for vesicles at *R =* 1.85, following the heating ramp (30 °C, 35 °C, 40 °C, and 30 °C after the transition).

According to polarized light microscopy results, as well as confirmed by the PGSTE analysis, no liquid crystals were present in any sample, where only one diffusion coefficient D was detected. Moreover, both micellar and monomeric dodecyl sulfate samples did not exhibit any difference in the attenuation slope, at different diffusion intervals Δ with an extremely fast exchange between the studied species and the bulk. Conversely, at different diffusion intervals Δ, the analysis of the diffusion coefficient D for dodecyl sulfate in vesicles showed a remarkable difference in the slopes, specifically between the Δ = 10 ms and Δ = 60 ms. Therefore, the exchange constant was found to be slow, in the range of few hertz. In the first stage of PGSTE experiments after the thermal transition, a Δ = 10 ms diffusion interval was applied to follow the vesicles’ thermal transition, with an *R* = 1.85 sample. Unfortunately, the spectrometer could not detect any changes before/after the thermal ramp with the mentioned experimental setup, whereas, at Δ = 60 ms, both a slightly faster exchange between the vesicular aggregates and the bulk and a slower echo decays were detected (Figure 7). Thus, a pseudo-phase transition (from multilamellar to unilamellar vesicles), involving thermodynamic changes in the system, took place [5].

**Figure 8:**
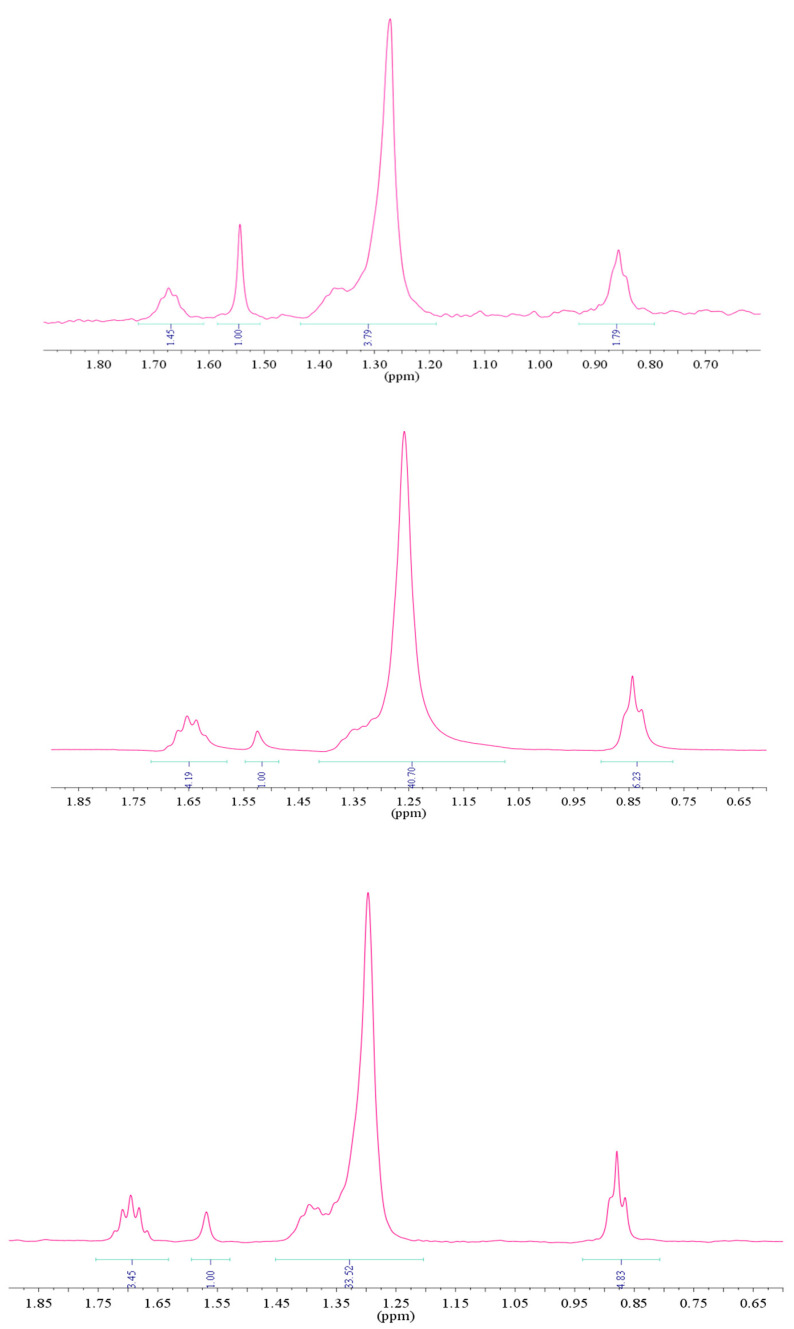
^1^H-NMR integrals comparison of aqueous solution related to (bottom to top) monomeric *SDS*, micellar *SDS*, and *SDS*/*CTAB* (*R* = 1.85) at 30 °C. Integral values were referred to as the water included in CDCl_3_.

Comparing ^1^H-NMR dodecyl sulfate integrals of monomeric (6 mM), micellar (10 mM), and vesicular solutions (3.9 mM for *R* = 1.85), it was possible to understand the relative populations in the vesicular aggregates (free component and included). A small amount of dodecyl sulfate in vesicular aggregates was available for detection, whereas the overall concentration of dodecyl sulfate was partitioned both in dodecyl sulfate monomeric (6 mM) and micellar (10 mM) forms, as per ^1^H-NMR integrals (Figure 8).

### 2.2. SDS/CPB Versus Sodium Decyl Sulfate (SdS)/CPB Systems

**Figure 9:**
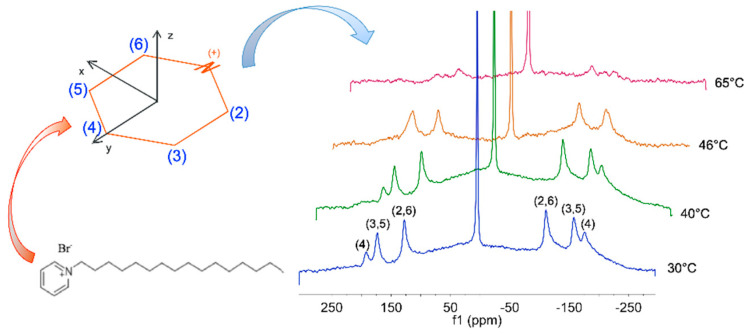
Clockwise, from left bottom to right. *CPB* molecular structure; pyridinium head group numbering and related axes for order parameters; 2H-NMR integrals comparison of SdS/*CPB* related to (bottom to top) 30 °C, 40 °C, 46 °C, and 65 °C. Signals corresponded to the fully deuterated head group of *CPB*. *CPB*, cetylpyridinium bromide; SdS, sodium decyl sulfate.

*SDS*/*CPB* (sodium dodecyl sulfate/cetylpyridinium bromide) and SdS/*CPB* (sodium decyl sulfate/cetylpyridinium bromide) were compared, taking advantage of the information by the pyridine substituent, to select the most appropriate and stable vesicular system. For this purpose, both anionic (vesicular systems) and cationic (mixed-micellar system) were changed. ^2^H-NMR was used to study a system with a molar ratio R = 1.7, composed of sodium decyl sulfate (SdS), cetylpyridinium bromide, with the fully deuterated head group (CPB-d_5_). The vesicular solution appeared milky, with no birefringence, as previously found for the *SDS/CTAB* systems. According to Figure 9, Δν(^2^H(2)) and Δν(^2^H(3)) showed a remarkable difference due to the geometry distortions from a regular hexagon of the pyridinium ring. The Δν(^2^H)s confirmed the order parameters—S_xx_, S_yy_, and S_zz_—of the pyridinium head-group, referred to the axes system. Moreover, a residual quadrupolar splittings Δν(^2^H(i)) for the “i” signal of the pyridinium ring in the ^2^H NMR spectrum was detected [24]. The isotropy of the solution was confirmed by the HOD resonance as a singlet, as previously found for the *SDS*/*CTAB* systems. The maxima in a Pake doublet corresponded to the equatorial orientation for a sphere.

**Figure 10:**
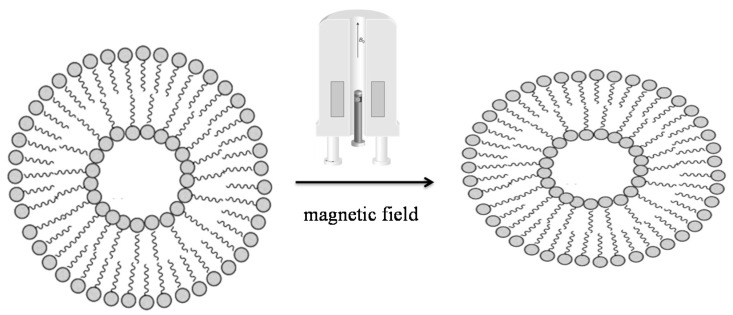
Representative scheme of the SdS/*CPB* vesicles deformation in the magnetic field.

Thus, the bilayers’ orientation as a consequence of a strong magnetic field exposure was proved by the deviations from the powder patterns, at the lowest temperature. Therefore, the results led to understand that ‘soft’ catanionic vesicles tended to an oblate shape (soft nanoonions), as previously found for the low elastic constant measured for *SDS*/*CTAB* vesicles, which showed to be able to easily undergo a shape deformation (Figure 10).

**Figure 11:**
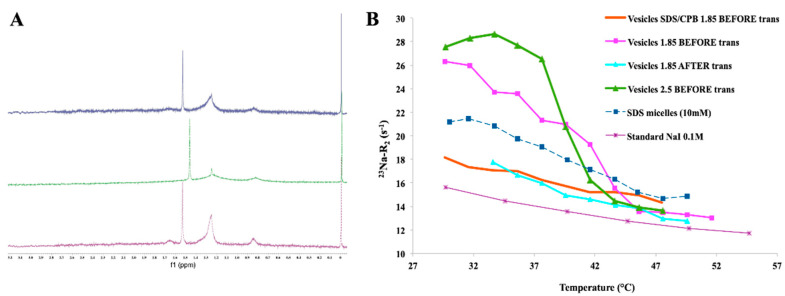
(**A**) ^1^H-NMR integrals of an *SDS*/*CPB* aqueous solution, bottom to top: 30 °C, 50 °C, 30 °C after transition; (**B**) ^23^Na-R_2_ values for 1.85 *SDS*/*CTAB* vesicular solution (before and after thermal transition), 1.85 *SDS*/*CPB* vesicular solution (before thermal transition), compared with a reference solution of NaI (free Na^+^) and a micellar solution of *SDS*.

Comparing the low elastic constant found for *SDS*/*CTAB* vesicles with the above-mentioned deformation, it was verified that they perfectly matched [8]. Indeed, the Δν(^2^H) values decreased with the temperature increase, as well as a remarkable broadening of the spectral lines. These data confirmed an exchange between the surfactant molecules of the vesicles and the monomers in bulk. With reference to previous observation on the tails in the catanionic system myristic acid-CTA, related to higher alkyl chain motions with a heating ramp via ^2^H-NMR, the mentioned trends looked opposite [25]. As per the reference system of *SDS*/*CTAB*, the ^1^H-NMR spectra of the *SDS*/*CPB* system confirmed that the only detectable component was the free dodecyl sulfate portion, in the exchange between the vesicles and the bulk. The ^23^Na-R_2_ trends on *SDS*/*CPB* system were quite similar to the *SDS*/*CTAB* system after the thermal transition, according to Figure 11.

### 2.3. Final Systems Optimization

Many surfactant mixtures were studied. Nevertheless, the most promising model appeared to be the one composed of *SDS* (sodium dodecyl sulfate) and *CTAB* (cetyltrimethylammonium bromide). Further investigation of other systems revealed that, e.g., *CPB* (cetylpyridinium bromide) tended to promote also crystals formation.

**Figure 12:**
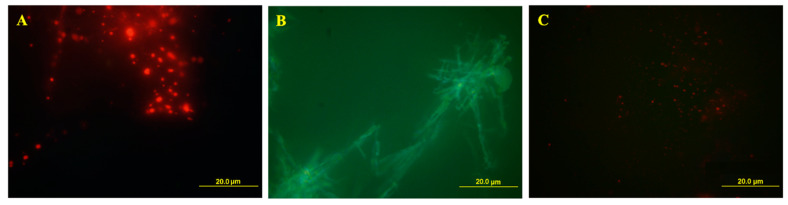
Fluorescence micrographs. (**A**) *SDS*/*CPB* system 24 h after preparation (vesicles, *R* = 1.85); (**B**) 1 month after preparation (crystals); all coming from the same solution. (**C**) *SDS*/*CTAB* vesicles system (*R* = 1.85), 24 h after preparation.

**Figure 13:**
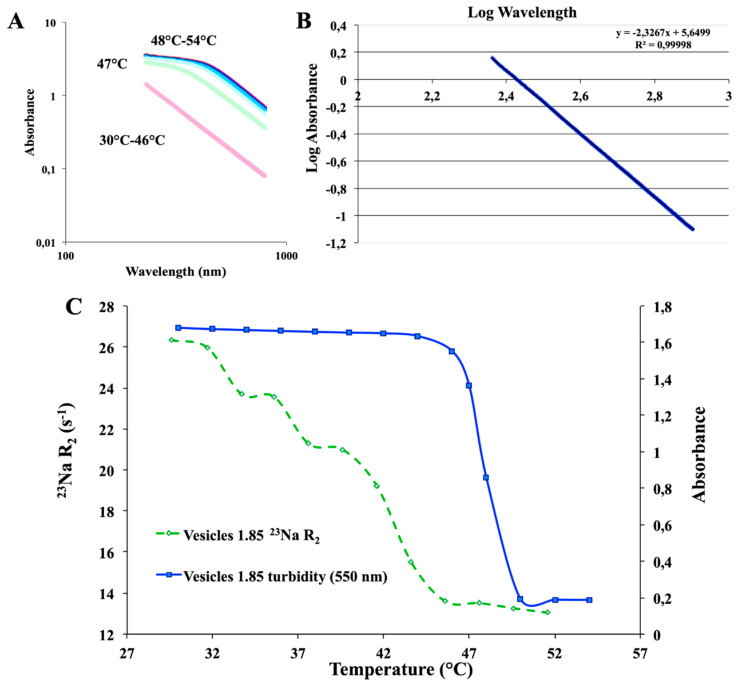
(**A**) UV-Vis spectra of an *SDS/CTAB* vesicles solution from 30 °C to 54 °C; (**B**) log/log plot of absorbance vs. wavelength; (**C**) comparison of turbidity and ^23^Na transverse relaxation rates of *SDS/CTAB* system.

Cetylpyridinium bromide was employed as the cationic counterpart to test the stability of the vesicular aggregates. In fact, as it possesses the lowest critical micellar concentration (CMC) of the considered surfactants, it was hypothesized that it could decrease the stability of the vesicular aggregates [18]. The *SDS*/*CPB* samples contained giant vesicles (diameter size of the order of 1 µm, see Figure 12), extremely polydispersed, whereas *SDS*/*CTAB* aggregates exhibited a lower dispersity, as well as smaller aggregates dimensions. In addition, CTAB was finally selected as the best cationic partner to optimize the most performing vesicular system. It led to samples with lower polydispersity, higher stability, and the best aggregates diameter [26]. Moreover, UV-Vis analyses were carried out (Figure 13) in the temperature range of 30 °C–54 °C, every 2 °C of the heating ramp, to completely characterize the system. This analysis revealed several interesting findings: (1) in a range of around λ ≈ 500–550 nm, “turbidity” was detected, (2) by plotting the obtained results on a log/log graph, a −2.3 slope was found, which correlated with a Mie scattering. This finding perfectly fitted with previous DLS dimensions referred to in the literature, which reports a particle size of approx. 500–550 nm [8]. Furthermore, all spectroscopy techniques (NMR and UV-Vis, Figure 13) looked to be suitable and sensitive to study and optimize the system.

## 3. Materials and Methods

### 3.1. Sample Preparation

Sodium dodecyl sulfate (SDS) was obtained from BDH Chemicals Ltd., Poole, England, UK (purity grade 99.0%), while cetyltrimethylammonium bromide (CTAB) was purchased from Sigma-Aldrich GmbH, Sternheim, Germany (purity ≥ 96%), as well as cetylpyridinium bromide (CPB), cetylbromide, and sodium decyl sulfate (SdS). Acridine orange (AO) was obtained from Sigma-Aldrich GmbH, Sternheim, Germany (purity ≥ 98%).

*CPB*-d_5_ was prepared by direct condensation of pyridine-d_5_ and cetylbromide in a boiling water bath (6 h). The product was crystallized from ethylacetate [24].

**Figure 14:**
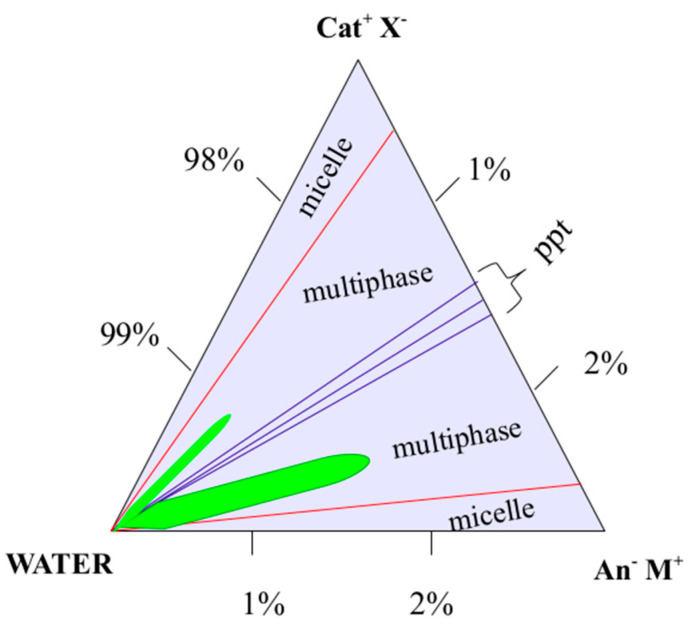
Phase diagram for the catanionic mixtures (vesicles formation in the green areas). “Ppt” stands for precipitation areas, “An” refers to the anionic partner, whereas “Cat” is the cationic component.

Aqueous solutions of CTAB, SDS, CPB, and SdS were prepared and subsequently mixed to obtain vesicular solutions. Different molar ratios (R) were employed, at a constant C_TOT_ = 6 mM (i.e., about 0.2 wt%), according to the phase diagram below (Figure 14). Vesicles were obtained by mixing the surfactant aqueous solutions at the molar ratio *R*, as per Equation (1), at 20 °C.

(1) $$R=\frac{\left[SDS\right]}{\left[CTAB\right]}\;R=\frac{\left[SDS\right]}{\left[CPB\right]}$$

### 3.2. Multinuclear NMR Experiments

NMR measurements were carried out on a Jeol Eclipse 400 NMR spectrometer (9.4 T), equipped with a Jeol NM-EVTS3 variable temperature unit (JEOL, Welwyn Garden City, UK), operating at 400 MHz for proton, 105.75 MHz for ^23^Na, 61.37 MHz for ^2^H, and 28.88 MHz for ^14^N. The measurements were carried out without field frequency lock, except in the case of the ^1^H spectra acquired with a lock on the signal of CDCl_3_ contained in a coaxial tube. The ^23^Na-R_2_ values (transverse relaxation rate, R_2_ = 1/T_2_) were measured by Hahn Echo [23].

### 3.3. PGSTE Measurements

The ^1^H NMR measurements were carried out on a Varian 500 MHz NMR spectrometer (11.74 T) operating at 500 MHz for ^1^H, equipped with a model L650 Highland Technology pulsed field gradient (PFA) amplifier (10 A) and a standard 5 mm indirect detection, PFG probe (Varian, Inc. Palo Alto, CA, USA). The lock was made on CDCl_3_ in a coaxial tube, containing tetramethylsilane (TMS) as ^1^H chemical shift reference. A one-shot sequence was employed for diffusion measurements [12,27], with 20 different z-gradient strengths, Gz, between 0.02 and 0.54 T/m, a pulsed gradient duration, δ, of 2 ms, and at different diffusion intervals (Δ). At each gradient strength, 64 transients were accumulated, employing a spectral width of 11 ppm over 16K data points. The solvent suppression was accomplished by presaturation. The gradients were calibrated on the value of D = 1.90·10^−9^ m^2^s^−1^ for ^1^H in D_2_O (99.9%) at 25 °C [28].

The values of the self-diffusion coefficient, D, were obtained by Tanner equation fitting to experimental data [29].

(2) $$\frac{E}{E_{0}}=\exp\left(-bD\right)\;b={\left(\gamma \delta G\right)}^{2}\left(\Delta -\frac{\delta }{3}\right)$$

where *E* and *E*_0_ are the signal intensities in the presence and absence of Gz, respectively, D is the diffusion coefficient, γ is the nuclear gyromagnetic ratio 26.75 × 10^7^ rad s^−1^ T^−1^ for ^1^H nucleus, δ is the gradient pulse width, G is the gradient amplitude, (Δ-δ/3) correspond to the diffusion time corrected for the effects of finite gradient pulse.

(3) q=γδG

PGSTE NMR spectra were processed using MestRenova, and the self-diffusion coefficients were determined by linear regression with Microsoft Excel.

### 3.4. UV-Vis

Spectral and absorbance measurements were carried out by using Shimadzu UV/Vis spectrophotometer model UV-2450 (Shimadzu, Tokyo, Japan), equipped with a Peltier temperature control unit; 1.0 cm path length matched quartz cells were used for the entire experimental work.

### 3.5. Polarized Light Microscopy

A Leitz Pol-Orthoplan microscope (Leitz GmbH, Wetzlar, Germany), equipped with differential interference contrast (DIC) lenses, was used. The main goal was to check the presence of crystals or anisotropic liquid crystals, such as lamellar phases, under polarized light. Samples were observed at room temperature, both immediately after preparation and several days later.

### 3.6. Confocal Microscopy

The integrity of catanionic vesicles was determined using acridine orange (AO) as the fluorescent probe. Samples were stained for 10 min with AO (0.3 mg/mL) and covered from sunlight. Immediately after, samples were examined using an Olympus microscope (Olympus, Tokyo, Japan), equipped with BX51M, a mercury UV-lamp (1000 W Ushio Olympus), with a set of filters of the type MNIBA3 (470 nm excitation and 505 nm dichromatic mirror). Subsequently, images were digitized through a video camera (Olympus DP70 digital camera) and analyzed with a raw image (Olympus DP Controller 2.1.1.176, Olympus DP Manager 2.1.1.158). All observations were made at 25 °C.

## 4. Conclusions

Several microscopic and spectroscopic techniques proved to be appropriate to characterize amphiphilic mixture systems. The heating ramp demonstrated to foster a remarkable Na^+^ dissociation, as the counterion of the excess surfactant (*SDS*), before the critical transition temperature [8]. Moreover, the bilayer of the pristine multilamellar vesicles exhibited an *SDS* dynamic release due to the temperature increase. When samples returned to the room temperature, vesicles demonstrated to be unilamellar, and a dynamic diffusion of dodecyl sulfate led to an increase of the vesicular aggregates curvature, boosting the ^23^Na transverse relaxation. Therefore, the above-mentioned curvature change was considered to be responsible for the stability at the high-temperature of the monolayer vesicular aggregates. Accordingly, an *SDS*/*CTAB* ratio raise occurred following the thermal ramp, as confirmed by ^1^H-NMR spectra. The multi-to-unilamellar transition was driven by a strong repulsion between the vesicular bilayers due to their surface net charge and the steric interactions.

In the optimization process, the application of *CPB* (cetylpyridinium bromide) as the cationic counterpart led to generate crystals; thus, it reduced the stability of vesicular aggregates. For what concerns the anionic partner, sodium decyl sulfate (SdS) proved to possess a very short alkyl chain, leading to excessive mobility of the monomers of the vesicular aggregates, as inferred by ^2^H-NMR in SdS/*CBP* samples, using *CPB* with the deuterated head group. As a confirmation of the observed phenomena, PGSTE experiments showed slower diffusion coefficients in vesicles after the thermal transition, as well as a slightly faster exchange between the vesicular aggregates and the bulk. Those findings endorsed a pseudo-phase irreversible thermal transition from multilamellar to unilamellar vesicles [5]. Hence, catanionic vesicles were optimized and revealed interesting temperature tunable changes both in their lamellar composition and curvature, depending on their composition and molar ratio.

Therefore, this kind of nanoreservoirs and their study by means of multinuclear NMR techniques propose an elegant approach to boost the ability of understanding and optimizing the encapsulation of a very small amount of bioactive molecules. Moreover, the present study provided for an in-depth awareness of all advantages that the optimized nanoreservoirs can broaden the applications’ plethora, such as the drug delivery sector. The catanionic vesicles preparation is quick and simple, the raw materials required are not expensive, and the nanoreservoirs can be used either on their multilamellar or their unilamellar form, thanks to their irreversible thermal transition. Furthermore, their soft nature allows them to include both hydrophilic and hydrophobic bioactive molecules.

## Figures and Tables

**Table 1:** Critical micellar concentrations (CMC) of the employed surfactants.

**Component**	**CMC**
Sodium decyl sulfate (SdS)	33 mM [16]
Sodium dodecyl sulfate (*SDS*)	8.3 mM [17]
Cetyltrimethylammonium bromide (*CTAB*)	0.93 mM [17]
Cetylpyridinium bromide (*CPB*)	0.33 mM [18]
**Component**	**CMC**
Sodium decyl sulfate (SdS)	33 mM [16]
Sodium dodecyl sulfate (*SDS*)	8.3 mM [17]
Cetyltrimethylammonium bromide (*CTAB*)	0.93 mM [17]
Cetylpyridinium bromide (*CPB*)	0.33 mM [18]
